# Next-Generation Sequencing Reveals a High Frequency of HIV-1 Minority Variants and an Expanded Drug Resistance Profile among Individuals on First-Line ART

**DOI:** 10.3390/v16091454

**Published:** 2024-09-13

**Authors:** Maria Nannyonjo, Jonah Omooja, Daniel Lule Bugembe, Nicholas Bbosa, Sandra Lunkuse, Stella Esther Nabirye, Faridah Nassolo, Hamidah Namagembe, Andrew Abaasa, Anne Kazibwe, Pontiano Kaleebu, Deogratius Ssemwanga

**Affiliations:** 1 Medical Research Council/Uganda Virus Research Institute & London School of Hygiene and Tropical Medicine, Uganda Research Unit, Entebbe P.O. Box 49, Uganda; maria.nanyonjo@mrcuganda.org (M.N.); dan.lule@mrcuganda.org (D.L.B.); nicholas.bbosa@mrcuganda.org (N.B.); sandra.lunkuse@mrcuganda.org (S.L.); stellaesther.nabirye@mrcuganda.org (S.E.N.); faridah.nassolo@mrcuganda.org (F.N.); hamidah.namagembe@mrcuganda.org (H.N.); andrew.abaasa@mrcuganda.org (A.A.); pontiano.kaleebu@mrcuganda.org (P.K.); deogratius.ssemwanga@mrcuganda.org (D.S.); 2 Uganda Virus Research Institute, Entebbe P.O. Box 49, Uganda; 3 Department of Molecular Biology, College of Veterinary Medicine, Makerere University, Kampala P.O. Box 7072, Uganda; anne.kazibwe@mak.ac.ug

**Keywords:** next-generation sequencing, HIV-1 antiretroviral therapy, minority variants, HIV-1 drug resistance

## Abstract

We assessed the performance and clinical relevance of Illumina MiSeq next-generation sequencing (NGS) for HIV-1 genotyping compared with Sanger sequencing (SS). We analyzed 167 participants, 45 with virologic failure (VL ≥ 1000 copies/mL), i.e., cases, and 122 time-matched participants with virologic suppression (VL < 1000 copies/mL), i.e., controls, 12 months post-ART initiation. Major surveillance drug resistance mutations (SDRMs) detected by SS were all detectable by NGS. Among cases at 12 months, SS identified SDRMs in 32/45 (71.1%) while NGS identified SDRMs among 35/45 (77.8%), increasing the number of cases with SDRMs by 3/45 (6.7%). Participants identified with, and proportions of major SDRMs increased when NGS was used. NGS vs. SS at endpoint revealed for NNRTIs: 36/45 vs. 33/45; Y181C: 26/45 vs. 24/45; K103N: 9/45 vs. 6/45 participants with SDRMs, respectively. At baseline, NGS revealed major SDRMs in 9/45 (20%) cases without SDRMs by SS. Participant MBL/043, among the nine, the following major SDRMs existed: L90M to PIs, K65R and M184V to NRTIs, and Y181C and K103N to NNRTIs. The SDRMs among the nine increased SDRMs to NRTIs, NNRTIs, and PIs. Only 43/122 (25.7%) of participants had pre-treatment minority SDRMs. Also, 24.4% of the cases vs. 26.2 of controls had minority SDRMs (*p* = 0.802); minority SDRMs were not associated with virologic failure. NGS agreed with SS in HIV-1 genotyping but detected additional major SDRMs and identified more participants harboring major SDRMs, expanding the HIV DRM profile of this cohort. NGS could improve HIV genotyping to guide treatment decisions for enhancing ART efficacy, a cardinal pre-requisite in the pursuit of the UNAIDS 95-95-95 targets.

## 1. Introduction

HIV, the causative agent of AIDS, still remains a global public health concern, with UNAIDS estimating that 38.4 million individuals across the globe were living with HIV in 2021 [1]. The WHO has a commitment to end this public health threat by 2030 through its 95-95-95 goals, which aim to have 95% of individuals living with HIV knowing their serostatus; 95% of those tested having access to HIV treatment; and 95% of them achieving virologic suppression by 2030 [2]. Achieving these ambitious targets requires concerted efforts in HIV/AIDS management strategies to ensure that treatment is not only accessible, but that its outcomes are sufficiently monitored. 

HIV antiretroviral therapy (ART) has remarkably improved HIV/AIDS treatment outcomes at both the individual and population level by reducing HIV-associated morbidity, mortality, and transmission, especially when virological suppression is attained. These crucial outcomes have inspired a massive global roll-out of ART and initiatives like test and treat. However, despite the gains registered so far from the rapid scale up of ART, the emergence and spread of HIV drug resistance (HIVDR) [3] associated with virologic failure (VF) are hampering the efforts in controlling the HIV epidemic. For this reason, drug resistance testing has become a standard procedure for the clinical management of the HIV/AIDS patients. Population sequencing using the SS method is used worldwide for HIVDR genotyping, a qualitative test that identifies mutations associated with HIVDR and reduced susceptibility of mutant HIV variants to ART drugs. However, the SS method cannot detect minority drug resistant variants with a frequency below 20% of the viral quasi-species [4,5,6]. Selection of minority variants due to ART drug pressure can make them the predominant variants [7]. 

Interest in minority HIV-1 drug-resistant variants is driven by the development of more sensitive and precise assays that can detect and quantify minority variants in large genetically complex populations of intra-host viruses. This is currently possible by use of next-generation sequencing (NGS) platforms that are available for the detection of minority drug-resistant variants [8]. The NGS platforms for deep sequencing, metagenomics, and whole-genome sequencing have become invaluable for identifying and characterizing viral pathogens, studying viral variations, enabling their accurate classification, and identification of viral genetic markers that correlate with virulence. Therefore, it is a suitable platform for surveillance, prevention, and control, as well as for the design of therapies for viral infections [9,10,11,12].

NGS platforms have been widely used to assess HIV viral diversity and minority drug resistance mutations. NGS assays have high sensitivity and offer a platform for high-throughput sequencing, enabling NGS to detect HIV minority variants that constitute as low as 0.05% to 20% of the HIV viral population [13,14,15]. Minority drug-resistance mutations (DRMs) are of clinical relevance since they can cause treatment failure in individuals initiated on HIV antiretroviral drugs [16,17,18,19]. Minority variants can reduce the efficacy of HIV drugs, for example, etravirine [20] and other NNRTI-based first-line regimens [21]. Treatment failure is when HIV ART regimen fail to control HIV infection, leading to virologic failure. In this study, treatment failure refers to virologic failure.

Most HIVDR studies carried out in Uganda have looked at major drug resistance mutations [22,23,24] with reliance on SS for HIV drug-resistance (HIVDR) genotyping. Although NGS promises to revolutionize HIV genotyping, its relevance in the clinical context has not been widely investigated. In Uganda, a few studies have used NGS in HIVDR genotyping: Using a deep sequencing platform, Kyeyune et al. [25] analyzed participants who were failing on ART that had no detectable DRMs by SS, and reported the existence of minority mutations that were associated with virologic failure and drug resistance. Recently, Ayitewala et al. [26] reported that the NGS-based in-house assay could be utilized for clinical HIVDR. In the current study, we assessed the performance of Illumina MiSeq NGS and compared the results to those previously obtained from SS [24]. To the best of our knowledge, this is the first study in Uganda to assess the performance of Illumina MiSeq NGS and to utilize it in HIV genotyping. Unlike most studies that only assess for HIVDR among viral failures, this study explored HIVDR among viral suppressors as well. We also determined the frequency of minority mutations in HIV-1-infected adults failing ART at 12 months post-ART initiation and, retrospectively, the mDRMs of their matched samples and controls at baseline. This study analyzed samples from a previous study in which we used a WHO HIVDR survey protocol and SS to assess acquired HIVDR among participants initiated on ART at three treatment centers from 2012 to 2013 [24]. In that survey, 20.9% of participants had major HIVDR, with the most prevalent mutations being M184V, Y181C, and K65R, as well as thymidine analogue mutations. Baseline viral load (VL) > 100,000 copies and CD4 count < 250 cells/μL were independent predictors of HIVDR [24].

## 2. Materials and Methods

**Figure 1:**
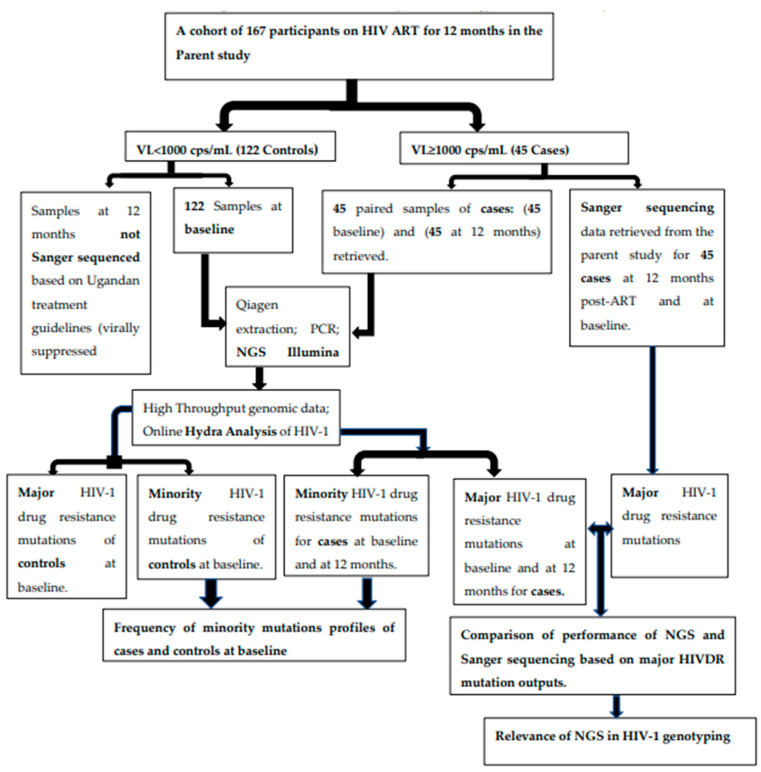
Study schema summarizing the key aspects of the study design and the major objectives.

Figure 1 summarizes the study methodology used in this study.

### 2.1. Study Design

This was a case–control study of HIV-1 ART-naïve participants who were initiated on ART and followed for 12 months after ART initiation. This study was nested in a parent observational cohort study conducted by the Ministry of Health, Uganda to profile acquired HIV-1 drug resistance after 12 months among individuals initiated on HIV-ART in three treatment centers in Masaka, Nsambya, and Mbale. A detailed description of the study site, study population, and sample collection in the parent study were published earlier on [24]. In this study, we used this cohort’s samples to assess the performance of the Illumina MiSeq NGS platform and determined the frequency of HIV-1 minority mutations. After 12 months on ART, we retrieved available samples of 45 participants with virologic failure (defined then in our setting as VL ≥ 1000 copies/mL) at the endpoint, who are cases in the current study. We randomly selected 122 participants with virologic suppression (VL < 1000 copies/mL) at both timepoints and designated them controls. Following the then Ugandan ministry of health HIV treatment guidelines requiring the HIV genotyping of only those with virologic failure [27], SS had been performed on the 45 samples of participants with virologic failure (VF) to identify HIV major DRMs at both baseline and at endpoint but HIV genotyping by SS had not been carried out for controls [24]. In this study, we performed NGS for 45 participants with VF at 12 months post-ART initiation (cases) to compare the major HIV surveillance DRMS outputs of NGS and compared with the SS output. Based on the variations in genotyping outputs of NGS and SS, we used the difference in genotyping outputs to explore the added value of NGS in HIV management. To determine and compare the profile of pre-treatment HIV minority mutations among cases and controls, we retrieved and performed NGS on 45 baseline (pre-treatment) samples of the cases and 122 baseline samples of controls. A summary of the study design and objectives is prese 

The dependent variable was HIV-1 genotyping performance of NGS in terms of the major SDRMs detected compared with the SS SDRMs for the same sequences at 12 months post-ART, and their respective baseline samples. To assess the clinical relevance of NGS, we examined the individual and public health implications of additional major SDRMs undetected by SS but detected by NGS. The other dependent variable was frequency of minority drug resistance mutations. A minority drug resistance mutation refers to any nucleotide change in the HIV-1 genome that changes the amino acid sequence of the wild-type virus, and this alteration is present in <20% of the viral population. Among the minority mutations are specific minority SDRMs that are the same as major mutations associated with treatment failure except that their frequency is <20% of the viral population. A major drug resistance mutation refers to any nucleotide change in the HIV-1 genome that changes the amino acid sequence of the wild-type virus, and this alteration is present in ≥20% of the viral population. Major HIV-1 SDRMs are those associated with treatment failure and reduced susceptibility of the virus to specific drug(s).

### 2.2. Laboratory Methods

#### 2.2.1. RNA Extraction, Amplification, and Detection of the Pol HIV Gene 

A total of 212 samples (45 samples before ART initiation, 45 samples after ART initiation, and 122 controls) were extracted and amplified as earlier described [24]. The SS assay was performed as earlier published [24,28].

#### 2.2.2. Library Preparation and NGS for the Detection and Quantification of Minority Resistant Variants

A detailed protocol for this work will be provided as Supplementary Material. We performed library preparation and NGS according to the methods earlier published [29]. The amplified PCR product of HIV-1 Pol gene (1.3 kb) from the protease (PR) and reverse transcriptase (RT) regions was cleaned using the Qiagen purification kit (Qiagen, Germany) according to the manufacturer’s instructions. We used 10 µL of the cleaned PCR amplicons for quantitation using the Qubit fluorometer (Invitrogen Thermo-scientific, Waltham, Massachusetts, USA) and the qubit ds DNA HS assay kit according to the manufacturer’s instructions. We prepared sequencing libraries using the Nextera XT DNA library preparation kit (Illumina, San Diego, CA, USA) according to the manufacturer’s protocol but after diluting the PCR products to 0.2 ng/µL. Library preparation included fragmentation based on transposon technology, then a PCR step incorporating dual indexes to the fragments and simultaneously tags the DNA with adaptor sequences. To ensure equal library representation during sequencing, library normalization was performed using the Nextera library normalization kit to obtain a 10-12 pM library with inserts that are 500–1000 base pairs. We diluted and pooled libraries prior to sequencing in the MiSeq Illumina platform. We used denatured Phix control (20 pM) from the Phix kit that was spiked at 20% in the pooled amplicons as a control.

In total, three MiSeq runs were performed. All the raw MiSeq data, obtained in FAST Q format, was processed using HyDRA web (https://hydra.canada.ca; accessed on 20 August 2021), a free pipeline for NGS-based HIVDR data analysis tool. HIVDR mutations detected above a 2% frequency were reported based on the default HyDRA Web Mutation Database, which is a combination of the Stanford 2015 list of HIV-1 drug resistance mutations (http://hivdb.stanford.edu; accessed on 20 August 2021), with added annotations from the WHO 2009 list of mutations for surveillance of transmitted HIVDR. HIVDR mutations were reported for reverse transcriptase and protease using the Stanford classification designations.

### 2.3. Data Collection, Processing, and Statistical Analysis

#### 2.3.1. Data Collection and Processing

Laboratory data for this study were collected between 2018 and December 2020. All Data were managed in Excel sheets, cleaned, and transferred to STATA version 15 (Stata Corp LP, College Station, TX, USA) for statistical analysis. 

The Illumina MiSeq platform generated FASTQ texts from nucleotide sequences along with matching quality scores. We analyzed the FASTQ files using HyDRA [30], an online-based pipeline. The HyDRA outputs included consensus sequences alongside an amino acid variant format (AAVF) file. The AAVF file had a summary of the amino acid variation translated from the NGS read pileup across the analyzed region of the HIV genome [31]. The AAVF files were uploaded to the Stanford University HIVDR Database for HIVDR profiling. 

#### 2.3.2. Statistical Analysis

In this study, Stata version 15 (Stata Corp LP, TX, USA) was used for statistical analysis and Microsoft Excel was used for graphs. Medians (interquartile ranges) were used for description of continuous variables. For categorical variables, proportions, frequencies, and percentages were used. Independent variables were compared between cases and controls using Chi-squares and the F-test where applicable. The Kruskal–Wallis test was used for comparison between groups. In all statistical analyses, only variables with *p* < 0.05 were reported as statistically significant and therefore independently associated with the dependent variable. Because of the skewed distribution, viral load was transformed on log base 10 scale. The HyDRA-generated reads were reported as estimated frequencies for both minority and major mutations. 

### 2.4. Ethical Considerations

Ethical approval was obtained from the Uganda Virus Research Institute (UVRI) Research and Ethics Committee and the Uganda National Council for Science and Technology (ref number: GC/127/15/12/203). All study subjects consented to the use of their samples for genetic studies and research.

## 3. Results

### 3.1. Study Cohort Demographic, Sampling, and Clinical Characteristics

**Figure 2:**
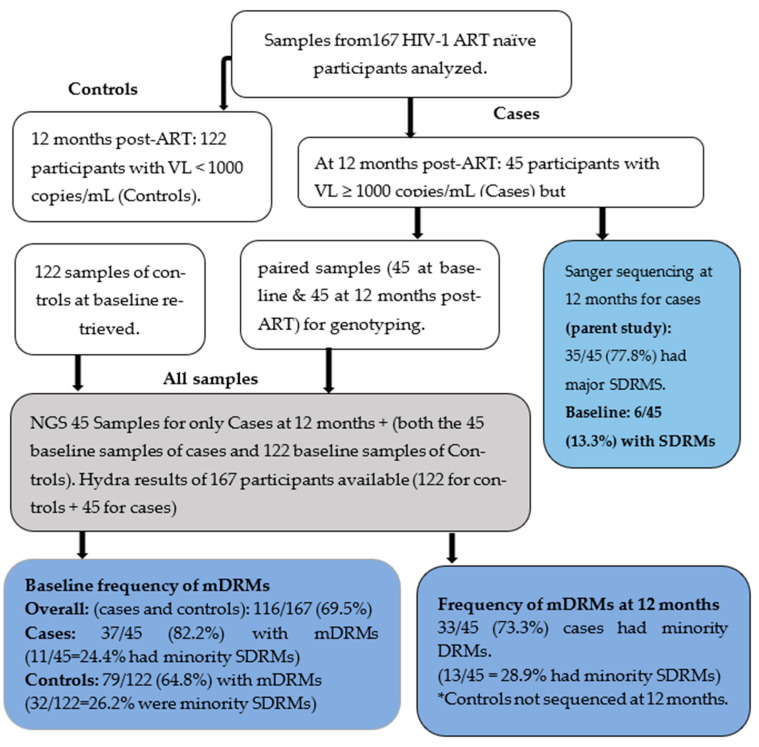
Summary of results. Samples from both groups (controls and the cases) were combined for HyDRA analyses, and the results in the last row are subdivided into baseline NGS results on the left (comprising baseline samples of controls and cases) and NGS results for only cases at 12 months. Controls were neither sequenced on the SS nor on the NGS platform at 12 months. Cases were those participants with virologic failure (VL ≥ 1000 copies/mL) at 12 months while controls were participants with virologic suppression (VL < 1000 copies/mL) at both 12 months and at baseline.

We analyzed 167 HIV-1-infected adults with a median age of 32.5 years (IQR: 26.5–39.5) who were initiated on ART. After 12 months on ART, 45 (16.9%) participants had failed to achieve virologic suppression, defined here as VL ≥ 1000 copies/mL. The individuals had been initiated on a first-line HIV ART regimen that by then constituted of a backbone of two NRTIs (either tenofovir (TDF) or azidothymidine (AZT)), plus either lamivudine (3TC) or emtricitabine (FTC), and one NNRTI, either efavirenz (EFV) or nevirapine (NVP). Among the 167, 52 (31.1%) were initiated on TDF+FTC+EFV/NVP, 55 (32.9%) were on TDF+3TC+EFV/NVP, and 60 (35.9%) were on AZT+3TC+EFV/NVP. Figure 2 summarizes the primary sequencing outputs of both the parent and the current study. 

### 3.2. Performance of NGS in HIV-1 Genotyping of 45 Participants with Virologic Failure at 12 Months Post-ART Initiation (Cases)

Among 45 participants with virologic failure at 12 months (cases), NGS detected HIV-1 major mutations among 35 (77.8%) participants. The participants had major mutations to NRTIs, NNRTIs, and PIs. Interestingly, NGS revealed the presence of major mutations in additional three participants in whom no major mutations were detectable by SS. Participants: MBL/031 (sequence EP_MBL-2200170) had major SDRMs to NRTIs, Y181C and K103N to NNRTIs; MBL/071 (sequence EP_MBL-2200204) had major SDMs M46I and L90M to PIs; and NSA/120 (sequence EP_NSA-2200215) had major SDRM K103N to NNRTIs. The matched outputs of NGS and SS for the 45 cases at 12 months post-ART initiation are presented in Table 1. The yellow highlights in red font show participants with major SDRMs only detected by NGS. The mutations in bold red were only detected by NGS. NGS detected major SDRMs among three participants who had no major SDRMs according to SS. 

#### 3.2.1. Comparison of Major SDRM Profiles Generated by NGS to Those from SS at 12 Months 

**Figure 3:**
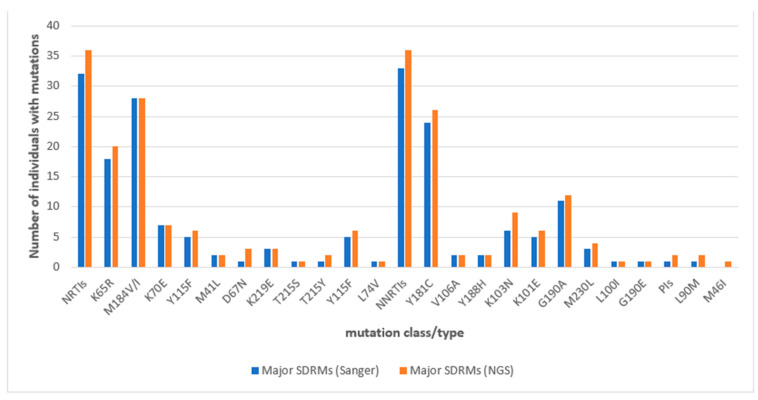
Comparison of major SDRM patterns based on SS and NGS genotyping for cases at 12 months: all major SDRMs detectable by SS were also detectable by NGS with the NGS platform detecting additional SDRMs reflected in the overlaps between the bars where they exist.

From Table 1, we observed that all major mutations detected by SS in participants’ sequences were detected by the Illumina MiSeq NGS platform showing 100% agreement (concordance). However, in some sequences, NGS detected additional major SDRMs to those earlier detected by SS. NGS detected major SDRMs in three participants where mutations were initially undetected by SS. The profiles of major SDRM patterns based on NGS and SS are represented in Figure 3. 

#### 3.2.2. Comparison of SS and NGS Major SDRM Outputs for Pre-Treatment Samples of Cases

**Figure 4:**
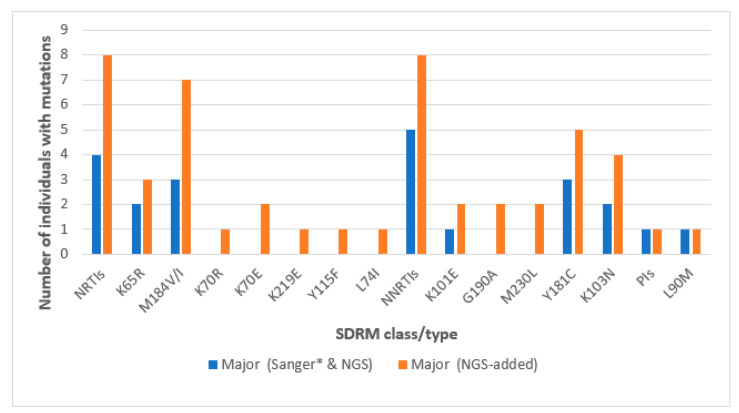
Major SDRMs detected by both SS and NGS (blue bars) and detected by only NGS (orange bars). * All major SDRMs detected by SS were detectable by NGS.

NGS detected major SDRMs in 15/45 (33.3%) baseline samples of cases. This was intriguing given that SS had detected major SDRMs in only 6/45 (13.3%), meaning NGS revealed major SDRMs among nine more participants (additional 20%) without SDRMs according to SS. Concerningly, one of the nine, participant MBL/043 (sequence BS_MBL-2200043), had SDRMs to all the three drug classes; L90M to PIs, K65R and M184V to NRTIs, and Y181C and K103N to NNRTIs. Six participants had both NRTIs and NNRTIs only, one participant had only one SDRM (G190A) to NNRTIs, and another participant had two SDRMs (M184I and K70E) to NRTIs (Table 2). The most prevalent NRTIs detected by NGS among the nine included M184V/I in seven, K65R in three, K70E in two participants, and K70R, L74I, Y115F, and K219E were present in one participant each. The most prevalent NNRTIs included Y181C in five, K103N in four, K101E in two, and G190A in two participants (Figure 4). 

#### 3.2.3. Proportions of Major SDRMs among Controls as Detected by NGS

**Figure 5:**
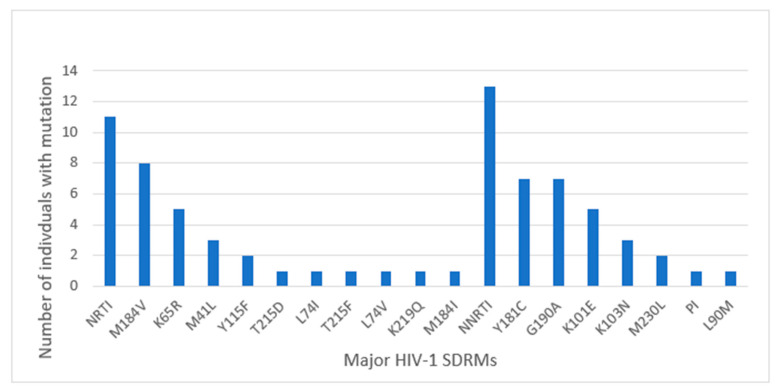
Major HIV-1 SDRMs detected among controls at baseline previously undetectable by SS.

Pre-treatment, controls were not genotyped, since they had VL < 1000 copies/mL and were not eligible for HIVDR testing. Among controls at baseline, NGS detected major SDRMs in 15/122 (12.3%) participants. The profile of major SDRMs detected is illustrated in Figure 5. 

#### 3.2.4. Proportions of Minority Drug Resistance Variants among Participants Experiencing Virologic Failure 12 Months Post-ART Initiation (Cases)

**Figure 6:**
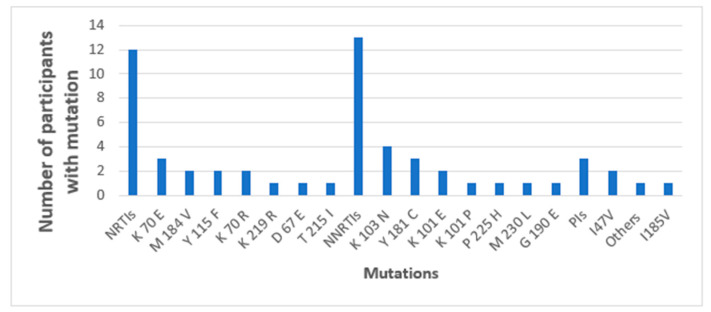
Minority drug resistance mutations of cases at 12 months post-ART initiation.

After 12 months on ART, 33/45 (73.3%) of the cases had minority DRMs; however, only 13/45 (28.9%) participants harbored minority SDRMs. The profiles of the mutation patterns of the cases at 12 months are summarized in Figure 6.

### 3.3. Proportions of Minority Drug Resistance among Participants at Baseline (Cases and Controls)

**Figure 7:**
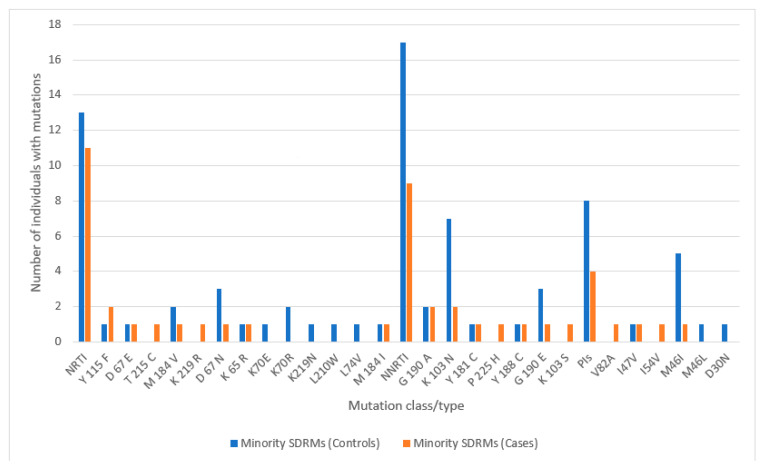
Minority drug resistance profiles of controls and cases at baseline.

Of the 167 (45 controls and 122 cases) participants with NGS results available, pre-treatment minority DRMs (irrespective of whether they are surveillance DRMs or not) were present in 116 (69.5%) of them. We detected surveillance pre-treatment minority SDRMs in 43/167 (25.7%) of both cases and controls. For the 45 cases before ART initiation, minority DRMs with a frequency range of 1.09–14.12%, were present in 37/45 (82.2%) of our participants (cases). But minority SDRMs existed in 11/45 (24.4%) of these cases. There were 11 participants with minority SDRMs to NRTIs and 9 participants with SDRMs to NNRTIs. The most frequent NRTI minority SDRM was present in two participants. Other NRTIs detected include M184V, D67E, and T215C, among others. Also, K103N and G190A, each of which were detected in two participants, were the most common NNRTI minority SDRMs. Four participants had PI minority SDRMs, such as V82A and M46I, among others (Figure 7). 

#### 3.3.1. Demonstrating the Clinical Relevance of NGS

In this study, we sought to demonstrate the clinical relevance of NGS based on additional major SDRMS and participants it detected with major SDRMs among for whom there were no detectable major SDRMs according to SS. NGS detected major SDRMs in 15/45 (33.3%) whereas SS had detected only 6/45 (13.3%) participants with major pre-treatment SDRMs. The addition of nine more participants (additional 20%) by NGS represents the potential added value of using NGS in HIVDR monitoring (Table 2). At baseline, 2/45 (4.4%) of our participants were revealed to harbor additional major SDRMs on top of those detected by SS. NGS detected major SDRMs K65R, D67N, K219E, and M184V to NRTIs, and K103N and Y181C to NNRTIs on top of L90M already detected by SS in participant MSK/053 (sequence BS_MSK_3300052). Participant NSA/042 (sequence BS_NSA-1100042) had major SDRMs K65R and M184V to NTRIs detected by NGS in addition to the K103N SDRM detected earlier by SS. We used these participants for demonstration but the actual picture of an expanded DRM profile following the use of NGS is broader and can be appreciated in Table 1 and Table 2. The clinical implications of an addition of a major SDRM to a participant’s profile can be illustrated with two participants in which NGS detected additional NRTIs and NNRTIs, respectively. For instance, participant BS_NSA-1100042 had increased resistance to all the NRTI drugs when NGS detected M184V and K65R major NRTI mutations. HIVDR score to ABC increased 60-fold, resistance to AZT by 25-fold, and to FTC/3TC by 90-fold (Table 3). Similarly, the detection of major NNRTI mutations K103N and G190A in the sequences of participant BS_MSK-3300054 increased resistance to DOR from 10 to 40, to EFV from 30 to 135, to ETR from 30 to 50, NVP from 60 to 180, and RPV from 45 to 70 (Table 4). 

The other added value of NGS in its ability to detect minority mutations whose relevance to clinical outcomes is still disputed. We sought to determine the relevance of the minority mutations in this study.

#### 3.3.2. Relevance of Pre-Treatment Minority Mutations on Treatment Outcomes

**Figure 8:**
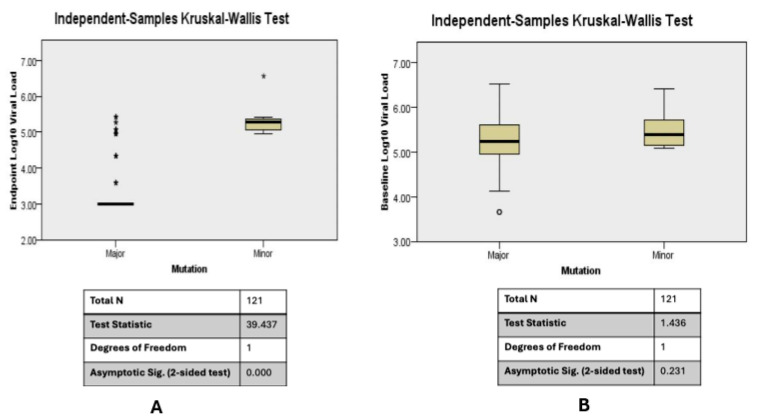
(**A**): Baseline HIV-1 drug resistance mutations plotted against endpoint viral load for both controls and cases show that the higher the frequency of baseline minority mutations, the higher the viral load count at the endpoint (Kruskal–Wallis *p* < 0.005). (**B**): Baseline HIV-1 viral loads are similar across timepoints for the cases and controls (Kruskal–Wallis, *p* = 0.231). In (**A**), The test statistic is adjusted for ties. Multiple comparisons were not performed since there were only two test fields. In (**B**), the test statistic is adjusted for ties. Multiple comparisons were not performed since the test showed no significant difference. The * (asterisk) are outliers above the fourth quartile and the small circle in (**B**) is an outlier below the l quartile.

**Figure 9:**
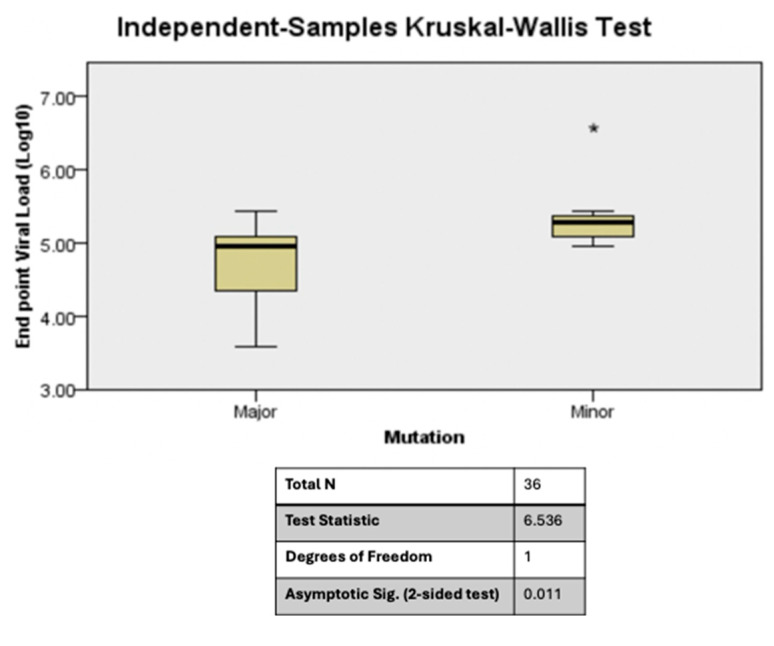
Baseline HIV-1 drug resistance mutations plotted against end point viral load for cases shows that the higher the frequency of minority mutations at baseline, the higher the viral load count at the endpoint (Kruskal–Wallis *p* = 0.011). The test statistic is adjusted for ties. Multiple regressions were not performed since there were only two test fields. The * (asterisk) represents outliers above the fourth quartile.

The presence of minority mutations at baseline correlated with higher viral loads after 12 months. For all samples (both controls and cases), the higher the frequency of minority mutations at baseline, the higher the viral load count at 12 months (Figure 8). One would probably hypothesize that this could have been due to variation in viral loads between the cases and controls at baseline; however, our analysis showed no significant difference between the mean (SD) count of viral load of cases (log5.4 (0.5)) and controls (log5.1 (0.77)) at baseline; *p* = 0.63. Even when cases at baseline were grouped into those with major and those with minority mutations, we noted that the higher the frequency of minority mutations, the higher the endpoint viral load count (Figure 9). These analyses were based on all minority mutations and not strictly minority SDRMs, which can become major SDRMs associated with treatment failure.

In Table 5, we summarized the proportions of those with pre-treatment minority SDRMs for the two groups, virologic failures (cases) and virologic suppressors (controls).

For baseline minority mutations to be relevant in virologic failure, the proportion of participants with baseline minority SDRMs should differ between the virologic failure group (cases) and the virologic suppression group (controls). We therefore tested the null hypothesis that the proportions of participants with pre-treatment minority mutations is the same in controls and cases. Pre-treatment minority SDRMs were present among 26.2% of participants in the virologic suppression group compared to 24.4% among the virologic failure group. Analysis based on the Z statistic and the two-tailed test determined the *p* value to be 0.802, which is greater than the 0.05 accepted level of significance. Therefore, there is a statistically significant difference in the proportions of participants with pre-treatment minority SDRMs between the controls and cases. This suggests that presence of minority SDRMs did not predict virologic failure at 12 months post-ART initiation. The factors associated with virological/treatment failure had been analyzed in the parent [24] study, except pre-treatment minority SDRMs. With our data suggesting no association between pre-treatment minority SDRMs and virologic failure, and having answered that objective in the parent study, we deemed it unnecessary to proceed with analysis of factors associated with virologic failure in the current study. 

## 4. Discussion

There is still a dearth of evidence with regard to the real clinical relevance of NGS for HIV-1 treatment monitoring in resource-constrained settings. This study assessed the clinical relevance of NGS in monitoring HIV treatment outcomes. We compared the HIV-1 genotyping performance of NGS with that of SS based on patterns of major and minority mutations. In this study, NGS detected all the major SDRMs earlier identified by SS, but it also identified additional SDRMS associated with HIVDR that were not detected by population SS. The presence of those mutations notably increased resistance to all the NRTI, NNRTI, and PI drugs. On a similar note, for participant MSK3300054, SS genotyping had only identified the NNRTI mutation Y181C. The NGS platform identified additional mutations K103N and G190A that increased the drug resistance scores to the existing NNRTI drugs. Studies have already reported that there is concordance between NGS and SS in terms of identifying mutations identified by SS [4,5,6,32]. The findings of this study agree with those of another study in which NGS identified additional mutations (to those identified by SS) that increased resistance to existing NRTI and NNRTI drugs [19]. 

We observed that the NGS platform expanded the drug resistance profile of some participants by identifying additional major SDRMs to those earlier detected by SS and this was observed at baseline (pre-treatment) and at 12 months post-ART initiation for cases. In addition, NGS identified additional participants with major SDRMs for whom SS had detected none. For instance, at baseline, 9/45 (20%) more cases had several SDRMs in addition to those revealed by SS; moreover, one of the nine had major SDRMs to NNRTIs, NRTIs, and PIs. While SS did not detect any SDRMs among controls at baseline, NGS intriguingly detected major SDRMs among 15/122 (12.3%) of the controls. This suggests that conventional population SS possibly misses some major HIV-1 SDRMs. This observation has serious ramifications on the HIV treatment at individual and population level. It means that even if SS had been carried out at baseline to inform treatment regimen for those participants, 15 of them would have been started on already failing regimens. The presence of participants with undetected major SDRMs endangers the public, as there is an increased possibility of transmission of resistant strains. We report an enhanced ability of NGS to detect mutations and identify participants with SDRMs undetectable to SS. This affirms to the greater sensitivity of NGS attributable to deep sequencing. These findings are in tandem with results from a survey that employed NGS in South America and concluded that reducing the variant detection threshold to 5% enhanced the identification of virologic failure among HIV-infected participants [33]. This further affirms to the benefits of NGS, that performs deeper and wider sequencing due to the parallel sequencing mechanism of the assay. 

Our findings further reiterate the importance of pre-treatment HIV-1 genotyping. We observed major pre-treatment SDRMs among both cases and controls, of which NGS revealed more participants with major HIV SDRMs than SS. Similarly, a survey in Uganda estimated the pre-treatment HIVDR at 18.2%, with NNRTI resistance being concerningly higher [28]. Also, pre-treatment HIVDR was associated with accumulation of DRMs and poor virological failure among Ugandans [34]. HIV genotyping prior to ART initiation is not recommended in the current Ugandan guidelines for HIV prevention and treatment [35]. Lack of pre-treatment HIV genotyping may inadvertently lead to increased cases with mutant HIV variants that are resistant to the initiated ART regimens, and eventually treatment failure, thus hampering global efforts to eradicate HIV. 

The new Ugandan guidelines have redefined virologic suppression as VL ≥ 201 copies/mL for plasma specimens and undetectable viral load for dried blood spot (DBS) specimens. However, HIV genotypic testing is only carried out after 12 months on ART for individuals with a persistent high viremia (VL ≥ 1000 copies/mL) after three sessions, after every two months, of intensive adherence counselling, CD4 monitoring, and repeat viral load testing [35]. In comparison, the European AIDS clinical guidelines take HIV-VL > 50 copies/mL after 6 months of HIV ART as virologic failure in those with previous undetectable viral load, and HIVDR genotypic testing is recommended for VL < 200–500 copies/mL [36]. Controls in this study who were participants with a VL < 1000 copies/mL at 12 months post-ART initiation and at baseline would not be eligible for HIVDR genotypic testing. Interestingly, with NGS analysis, major NNRTI, NRTI, and PI drug resistance mutations were detected in 11, 13, and 1 participant(s), respectively. Since VL test results are used to guide on who should be subjected to genotypic resistance testing, the VL ≥ 1000 copies/mL used to qualify one for HIV genotyping needs to be reduced. In developed settings, the VL threshold for virologic failure is more stringent; for example, in Europe, a threshold VL of >50–200 copies/mL defines virologic failure [36]. In agreement with our findings, some researchers have already observed treatment failure in form of drug resistance among virologically suppressing participants with a VL < 1000 copies/mL [25,37]. Therefore, some individuals with major HIVDR may be left out of the necessary HIV genotypic resistance testing on the assumption that those with a VL < 1000 copies/mL are less likely to have HIV DRMs. 

Our NGS assay detected pre-treatment minority variants among 64.7% of our study participants. This is a similar frequency to that obtained by Clutter et al. [38], where they detected minority DRMS in 60% of the study participants. The frequency reported in this study is however lower than the 80% prevalence obtained in a Malawian study [19]. The current study provides sufficient evidence that NGS can detect minority mutations that are often missed out by the most widely used population SS. This finding concurs with the reports of several studies that have credited NGS with the ability to detect low-frequency HIVDR variants [19,25,38,39,40,41].

HIV-1 minority variants (also referred to as low-frequency mutations), though undetectable to the conventional population SS, are said to be clinically crucial, as they have been associated with virologic failure in participants initiated on ART [8,25]. Specific HIV-1 variants that are clinically significant at a level as low as 1% of the viral population may replicate quickly and become the major viral population due to selective pressure of ART drugs, resulting in treatment failure [41]. We detected the most common NRTI minority mutations as M184V/I, Y115F, and D67E, while the most prevalent NNRTI minority mutations included K103N, Y181C, and G190A. These findings mirror those that were reported in Malawi among participants on an ART regimen composed of NRTIs and one NNRTI [19]. In the current study, NGS detected PI mutations that had not been detected by SS. The common PIs detected by NGS included M46I/L, D30N, I47V, and V82A. Zhou et al. [19] reported the detection of PIs that SS did not reveal. Since the PIs were not yet being used in this population at the time of sample collection, the PI mutations detected by NGS here could be natural polymorphs of HIV-1. It is, however, concerning, as natural polymorphisms of M46L have been shown to have a replicative advantage for HIV-1 subtype B [42]. As Uganda has included the use of PIs for second-line regimens, the presence of PI minority mutations should be closely monitored to prevent exacerbation of drug resistance to this class of salvage therapy.

Kyeyune et al. [25] previously observed that some adhering participants continued to fail on ART and yet SS revealed no HIV-1 drug resistance mutations. This suggests that SS could have possibly missed out on some HIV mutations (probably minority mutations with clinical relevance), and therefore points to the need to use more robust deep sequencing platforms to enhance HIV treatment monitoring and guide treatment decisions. In addition, the NGS platform also revealed minority DRMS among 79/122 (64.8%) of the controls in this study. These mutations spanned across all the three drug classes of NRTIs, NNRTIs, and PIs. The minority DRMs observed here are probably a mixture of transmitted drug resistance mutations that faded to frequencies undetectable by SS and de novo mutations arising from poor incorporation and high error rate of HIV-1 transcriptase enzyme [38]. The NGS analysis of samples of controls that were collected at baseline revealed the presence of minority variants. Without drug pressure, resistant virus populations (variants) are selected against, as they are outmatched by wild-type variants that have more efficient replication ability [43]. This results in resistant variants having low frequencies (below 20%), which cannot be detected by population SS, but are detected by NGS [43,44]. 

While the actual clinical relevance of minority DRMS remains contested [32,43,44], other studies [19,21,25,38] have reported association between minority variants with virologic failure. Zhou et al. [19] analyzed minority HIV resistance in a Malawian cohort and observed that minority mutations increased the resistance levels of HIV to, not only some of the NRTIs and NNRTIs used, but also to future possible salvage regimen of the same of NNRTIs and NRTIs. The implication of this is that minority HIV-1 drug resistance mutations could possibly hamper HIV treatment efforts by reducing the efficacy of the possible future regimen. That could in turn prevent the realization of the UNAIDS goal to eliminate the public threat posed by HIV by 2030, which requires the achievement of the 95-95-95 targets. In our study, there was no statistically significant difference in the proportion of participants with pre-treatment minority SDRMs between controls and cases (*p* = 0.802), suggesting that pre-treatment minority SDRMs are non-predictors of virologic failure. This result could be so because we based our analyses on only pre-treatment minority SDRMs and not all minority mutations detected by NGS. Virologic failure among our cases could be due to additional major SDRMs that were undetectable by SS but were detected by NGS. 

Our study is among a few studies in Uganda that have evaluated the utilization of NGS for HIV drug HIVDR genotyping, the other having been conducted by Kyeyune et al. [25], and a recent validation study by Ayitewala et al. [26]. This report adds to the limited data on minority HIV-1 drug resistance in our region and provides insights into the potential relevance of NGS in HIVDR testing. We had a sufficient sample size that included both cases and controls, which augments our findings. Also worth noting are the sequencing data that were available at both baseline and time of virologic failure, which enable comparison of the NGS platform output with that of SS.

However, the findings of this study should be interpreted with the consideration that the samples were collected in 2014 when participants were on some of the HIV-1 ART drugs that are now not being used. The Ministry of Health has updated its treatment guidelines, and some NNRTI drugs like NVP have been discontinued and replaced with dolutegravir as an anchor to two NRTIs, usually tenofovir and lamivudine (the common adult first-line HIV ART regimen combination now includes TDF+3TC+DTG) [35]. Also, the definition of virologic failure/non-suppression has now changed to low viremia non-suppression (plasma VL ≥ 201 copies/mL) and to high viremia non-suppression (VL ≥ 1000 copies/mL). Although samples of virologic failures were genotyped at 12 months, samples of virologic suppressors were not subjected to NGS analyses at 12 months. It is possible that useful insights that could have arisen from such a comparison may have been missed. In addition, being a case–control study, the frequencies of virologic failure and suppression cannot be regarded as a true value of prevalence. The controls were selected to match cases, and the sample population used may not be truly representative of the general population in this setting.

## 5. Conclusions

NGS detected all major SDRMs earlier detected by SS, but NGS detected additional major SDRMs and identified more participants harboring major SDRMs than SS, expanding the HIV DRM profile of this cohort. Pre-treatment (baseline) major SDRMs were reported by both platforms among cases but the robust NGS reported even more. Our data thus suggest that HIV major SDRMs earlier undetected by SS but detectable by NGS partly contributed to virological failure in this cohort. The sequencing depth of NGS revealed a high frequency of minority mutations but with a low proportion of pre-treatment minority SDRMs that were not associated with virological failure. NGS, being a robust and deep sequencing platform, could improve HIV genotyping to guide treatment decisions for enhancing ART efficacy, a cardinal pre-requisite in the pursuit of the UNAIDS 95-95-95 targets.

## Figures and Tables

**Table 1:** HIV-1 SDRM outputs based on SS and NGS genotyping of 45 cases at 12 months.

Sequence ID	PID	PI. SDRMs		NRTI. SDRMs		NNRTI. SDRMs	
		SS	NGS	SS	NGS	SS	NGS
EP_MBL-2200184	MBL/017	none	none	K65R, M184V	K65R, M184V,	V106A, Y181C	V106A, Y181C
EP_MBL-2200162	MBL/030	none	none	none	none	Y188HL	Y188HL
EP_MBL-2200170	MBL/031	none	none	none	**K65R**	none	**Y181C, K103N**
EP_MBL-2200237	MBL/043	none	none	K65R, M184V	K65R M184V	V106M	V106M
EP_MBL-2200213	MBL/025	none	none	M184V	M184V	none	None
EP_MBL-2200242	MBL/053	none	none	none	none	K103N, Y188H	K103N Y188H
EP_MBL-2200204	MBL/071	none	**M46I, L90M**	none	none	none	none
EP_MBL-2200243	MBL/096	none	none	none	none	G190S	**K103N**, G190S
EP_MBL-2200194	MBL/102	none	none	M184V	M184V, **T215Y**	K101E, G190A	K101E, G190A, **Y181C**
EP_MBL-2200257	MBL/137	none	none	M184V	M184V/I	K103N	K103N, **Y181C, K103S**
EP_MBL-2200238	MBL/143	none	none	M184V	M184V	Y181C, G190A	Y181C, G190A,
EP_MSK-2200233	MSK/005	none	none	K65R, M184IV	K65R, M184V/I	K103N, Y181C	K103N, Y181C, **G190A**
EP_MSK-2200218	MSK/017	none	none	K70E, Y115F, M184IV	K70E, Y115F, M184IV	Y181C	Y181C
EP_MSK-2200174	MSK/017	none	none	K65R, M184I	K65R, M184I	Y181C, M230L	Y181C, M230L
EP_MSK-2200194	MSK/024	none	none	none	none	none	none
EP_MSK-2200201	MSK/034	none	none	K70E, M184I	K70E, M184I	K103S, Y181C	K103S, Y181C
EP_MSK-2200182	MSK/038	none	none	K70E, M184V	K70E, M184V/I	Y181C, G190A	Y181C, G190A, **K101E**
EP_MSK-2200186	MSK/053	L90M	L90M	K65R, M184IV	K65R M184IV	K103N, Y181C	K103N Y181C
EP_MSK-2200165	MSK/055	none	none	M41L, K65R, M184V	M41L, K65R M184V, **D67N**	Y181C, G190A	Y181C, G190A **K219KE**
EP_MSK-2200210	MSK/061	none	none	K65R, Y115F, M184V	K65R, Y115F, M184V	Y181C	Y181C, **M230L**
EP_MSK-2200219	MSK/074	none	none	K65R, D67N, M184V, K219E	K65R, D67N, M184V, K219E	K103N, Y181C	K103N, Y181C, **M230L**
EP_MSK-2200161	MSK/040	none	none	K65R, M184I	K65R, M184V/I	K101E, Y181C, G190A, M230L	K101E, Y181C, G190A, M23OL
EP_MSK-2200178	MSK/089	none	none	K70E, M184V/I	K70E, M184V/I	K101E, Y181C, G190A	K101E, Y181C, G190A
EP_MSK-2200227	MSK/095	none	none	K65R, Y115F	K56R, Y115F	L100I, Y188C	L100I, Y188C
EP_MSK-2200190	MSK/097	none	none	K70E, M184I	K70E M184I	Y181C	YI181C
EP_MSK-2200244	MSK/102	none	none	none	none	none	none
EP_MSK-2200214	MSK/122	none	none	K70E, M184V	K70E M184V	Y181C	Y181C
EP_MSK-2200221	MSK/123	none	none	M184I	M184I, **K170E**	Y181C	Y181C
EP_MSK-2200261	MSK/125	none	none	T215S	T215S	none	none
EP_MSK-2200211	MSK/126	none	none	none	none	none	none
EP_MSK-2200217	MSK/135	none	none	K65R	K56R	Y181C, G190A	Y181C, G190A
EP_NSA-2200157	NSA/002	none	none	K65R, Y115F, M184V	K65R Y115F M184V	Y181C, G190A	Y181C G190A
EP_NSA-2200190	NSA/003	none	none	K65R, M184V	K65R M184V	K103N, Y181C	K103N Y181C
EP_NSA-2200218	NSA/015	none	none	M184V, T215Y	M184V, T215Y	K101E, Y181C, G190A	K101E, Y181C G190A
EP_NSA-2200248	NSA/016	none	none	none	none	V106A	V106A
EP_NSA-2200165	NSA/029	none	none	D67N, K70E, M184IV, K219E	D67N. K70E. M184V, K219E	G190E	G190E
EP_NSA-2200183	NSA/042	none	none	none	**K65R, Y115F,** **M184V**	none	**Y181C**
EP_NSA-2200223	NSA/076	none	none	K65R, M184V	K65R M184V	Y181C	Y181C
EP_NSA-2200262	NSA/077	none	none	none	none	none	none
EP_NSA-2200228	NSA/079	none	none	M41L, K65R, M184V	M41L, K65R, M184V	K101E, Y181C, G190A	K101E, YI81C, G190A
EP_NSA-2200204	NSA/085	none	none	K65R, M184I	K65R M184I	Y181C, M230L	Y181C M230L
EP_NSA-2200209	NSA/111	none	none	K65R, M184V, K219E	K65R, M184V, K219E	none	none
EP_NSA-2200222	NSA/112	none	none	K65R, L74V, Y115F, M184I	K65R, Y115F, M184I, L74V	Y181C, G190A	Y181C G190A
EP_NSA-2200215	NSA/120	none	none	none	none	none	**K103N**
EP_NSA-2200261	NSA/130	none	none	none	none	none	none

Note: The SDRMs in bold texts were only detected by NGS. NGS detected major SDRMs among three participants who had no major SDRMs according to SS.

**Table 2:** Comparison of SS and NGS genotyping outputs for pre-treatment samples of cases.

Sequence ID	PID	PI. SDRMs		NRTI.SDRMs		NNRTI.SDRMs	
		SS	NGS	SS	NGS	SS	NGS
BS_MBL-2200017	MBL/017	none	none	none	**K70E, M184I**	none	none
BS_MBL-2200030	MBL/030	none	none	none	none	none	none
BS_MBL-2200031	MBL/031	none	none	none	none	none	none
BS_MBL-2200043	MBL/043	none	**L90M**	none	**K65R, M184V**	none	**K103N,** **Y181C**
BS_MBL-2200052	MBL/025	none	none	none	none	none	none
BS_MBL-2200053	MBL/053	none	none	none	none	none	none
BS_MBL-2200071	MBL/071	none	none	none	none	none	none
BS_MBL-2200096	MBL/096	none	none	none	none	none	none
BS_MBL-2200102	MBL/102	none	none	none	none	none	none
BS_MBL-2200137	MBL/137	none	none	none	none	none	none
BS_MBL-2200143	MBL/143	none	none	none	**M184I, K65R**	none	**Y181C,** **M230L**
BS_MSK-3300005	MSK/005	none	none	none	none	none	none
BS_MSK-3300015	MSK/017	none	none	none	none	none	none
BS_MSK-3300016	MSK/017	none	none	none	none	none	none
BS_MSK-3300024	MSK/024	none	none	none	none	none	none
BS_MSK-3300034 *	MSK/034	none	none	none	none	K103N	K103N
BS_MSK-3300038	MSK/038	none	none	none	none	none	none
BS_MSK-3300052 *	MSK/053	L90M	L90M	none	**K65R, D67N,** **K219E, M184V**	none	**K103N, Y181C**
BS_MSK-3300054 *	MSK/055	none	none	E44D; M184V	E44D; M184V	Y181C	Y181C
BS_MSK-3300060	MSK/061	none	none	none	none	none	none
BS_MSK-3300073	MSK/074	none	none	none	none	none	**G190A**
BS_MSK-3300074	MSK/075	none	none	none	**M184V**	none	**K101E, M230L**
BS_MSK-3300088	MSK/089	none	none	none	none	none	none
BS_MSK-3300094	MSK/095	none	none	none	none	none	none
BS_MSK-3300095	MSK/096	none	none	none	none	none	none
BS_MSK-3300097	MSK/098	none	none	none	none	none	none
BS_MSK-3300121	MSK/122	none	none	none	none	none	none
BS_MSK-3300122	MSK/123	none	none	none	none	none	none
BS_MSK-3300124 *	MSK/125	none	none	T215S	T215S	none	none
BS_MSK-3300125	MSK/126	none	none	none	none	none	none
BS_MSK-3300134	MSK/135	none	none	none	none	none	none
BS_NSA-1100002	NSA/002	none	none	none	none	none	none
BS_NSA-1100003	NSA/003	none	none	none	**K65R, M184V**	none	**K103N, Y181C**
BS_NSA-1100015 *	NSA/015	none	none	T215D	T215D,	K101E, Y181C	K101E, Y181C
BS_NSA-1100016	NSA/016	F53L	none	none	none	none	none
BS_NSA-1100029	NSA/029	none	none	none	**K70R, K219E**	none	**Y181C**
BS_NSA-1100042 *	NSA/042	none	none	none	**K65R, M184V**	K103N	K103N
BS_NSA-1100076	NSA/076	none	none	none	**L74I, Y115F, M184V**	none	**K103N**
BS_NSA-1100077	NSA/077	none	none	none	none	none	none
BS_NSA-1100079	NSA/079	none	none	none	none	none	none
BS_NSA-1100085	NSA/085	none	none	none	none	none	none
BS_NSA-1100111	NSA/111	none	none	none	**K70E, M184V,**	none	**K101E, Y181C, G190A**
BS_NSA-1100112	NSA/112	none	none	none	none	none	none
BS_NSA-1100120	NSA/120	none	none	none	none	none	none
BS_NSA-1100130	NSA/130	none	none	none	none	none	none

Note: * represents Participants whose SDRMs were detected at baseline by SS. The SDRMs in bold texts were detected by only NGS.

**Table 3:** Comparison of clinical effect of NRTI mutations based on SS and NGS outputs of cases at baseline.

Id	Mutations by SS	GSS of Drugs	Mutations by NGS	Gss of Drugs
**BS_MSK-** **3300054**	E44D; M184V	ABC (15); AZT (1); D4T (−10); FTC (60); 3TC (60); TDF (−10)	E44D; M184V	ABC (15); AZT (1); D4T (−10); FTC (60); 3TC (60); TDF (−10)
**BS_MSK-** **3300124**	T215S	ABC (15); AZT (20); D4T (20); FTC (0); 3TC (0); TDF (5)	T215S	ABC (15); AZT (20); D4T (20); FTC (0); 3TC (0); TDF (5)
**BS_NSA-** **1100042**	None	ABC (0); AZT (0); D4T (0); FTC (0); 3TC (0); TDF (0)	M184V; K65R	**ABC (60); AZT (−25);** **D4T (50); FTC (90);** **3TC (90); TDF (50)**

**Table 4:** Comparison of clinical effect of NNRTI mutations based on SS and NGS outputs of cases at baseline.

Id	Mutations by SS	GSS of Drugs	Mutations by NGS	GSS of Drugs
**BS_MSK-3300054**	Y181C	DOR (10); EFV (30); ETR (30); NVP (60); RPV (45)	Y181C; K103N; G190A	**DOR (40);** **EFV (135);** **ETR (50);** **NVP (180);** **RPV (70)**
**BS_MSK-3300124**	None	DOR (0); EFV (0); ETR (0); NVP (0); RPV (0)	None	DOR (0); EFV (0); ETR (0); NVP (0); RPV (0)
**BS_NSA-1100042**	K103N	DOR (0); EFV (60); ETR (0); NVP (60); RPV (0);	K103N	DOR (0); EFV (60); ETR (0); NVP (60); RPV (0);

**Table 5:** Contingency table showing proportions of participants with pre-treatment minority mutations among controls and cases.

	Pre-Treatment Minority SDRMS Present	Pre-Treatment Minority SDRMs Absent	Total
Virologic suppressed group (controls)	32 (26.2%)	90 (73.8%)	122
Virologic failure group (cases)	11 (24.4%)	34 (75.6%)	45
**Total**	43 (25.7%)	124	167

## Data Availability

The datasets generated and analyzed during the current study are not publicly available due to restriction policies and data protection policies of the MRC/UVRI and LSHTM but can be made available by the corresponding authors on reasonable request and on approval of the UVRI Research Ethics Committee.

## Supplementary Materials

The following supporting information can be downloaded at: https://www.mdpi.com/article/10.3390/v16091454/s1, Document S1: A detailed laboratory protocol.
