# Supplementary material for: Next-Generation Sequencing Reveals a High Frequency of HIV-1 Minority Variants and an Expanded Drug Resistance Profile among Individuals on First-Line ART
Source: Viruses. 2024 Sep 13;16(9):1454. doi: 10.3390/v16091454 (PMC11437406; doi:10.3390/v16091454)
Supplement: Supplementary file 1 [file viruses-16-01454-s001.zip › viruses-3087993-supplementary.pdf]

SUPPLEMENTARY FILE S1

Detailed Protocol of Laboratory Methods

A schematic presentation of the entire workflow

The laboratory methods that were employed to achieve the objectives set by this study are summarized in figure S1.

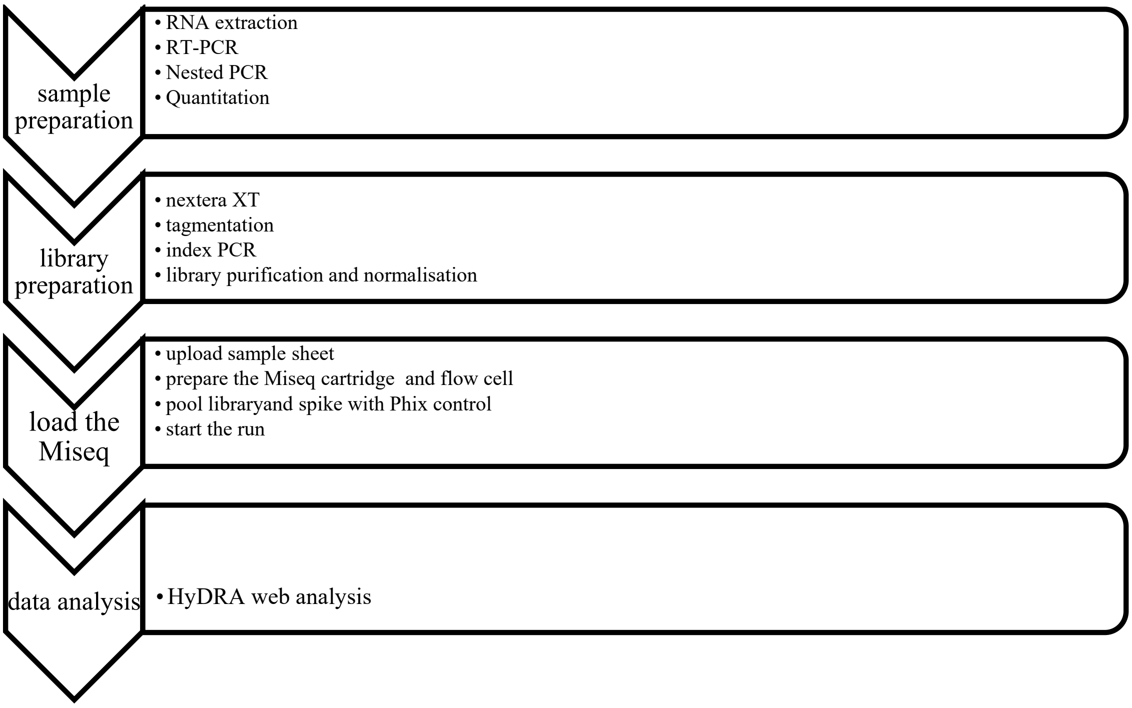

Figure S1: Laboratory workflow

Following unidirectional workflow, samples are extracted, reverse transcribed before libraries are prepared and then loaded to the Miseq. Data analysis to obtain resistant variants was done using a web- based tool called HyDRA.

RNA extraction, amplification, and detection of the pol HIV gene

RNA extraction was carried out on a total of 212 plasma samples (45 samples before ART initiation, 45 samples after ART initiation and 122 controls). The samples were genotyped in the WHO designated laboratory at MRC/UVRI/LSHTM-Entebbe. RNA was successfully extracted from 140µl of plasma using Qiagen viral RNA extraction kit protocol (Qiagen, Hilden, Germany). 560 µl of Buffer AVL containing carrier RNA was pipetted into a 1.5-ml micro centrifuge tube.

140 µl plasma was added to the Buffer AVL/Carrier RNA in the micro centrifuge tube. This was mixed by pulse-vortexing for 15 seconds and incubated at room temperature (15-25°C) for 10 minutes to ensure efficient viral particle lysis. 560 µl of ethanol (96 -100%) was added to the lysed sample and mixed by pulse-vortexing for 15 sec. 630 µl of the solution was poured on to the QIAamp Mini spin column (in a 2 ml collection tube) without wetting the rim and centrifuged at 6000 x g (8000 rpm) for 1 minute. Then the QIAamp spin column was placed into a clean 2-ml collection tube, and the tube containing the filtrate was discarded. The process was repeated until all the solution was finished from the micro centrifuge tube. 500 µl of Buffer AW1 was added to the QIAamp Mini spin column and centrifuged at 6000 x g (8000 rpm) for 1 minute. The QIAamp Mini spin column was placed into a clean 2 ml collection tube and the tube containing the filtrate was discarded. 500 µl of Buffer AW2 was added to spin column and centrifuged at full speed (20,000 x g; 14,000 rpm) for 3 minutes. The QIAamp Mini spin column was placed into a clean 1.5 ml micro centrifuge tube. 60 µl of Buffer AVE was placed into the QIAamp spin column and incubated at room temperature for 1 minute. This was finally centrifuged at 6000 x g (8000 rpm) for 1 minute. The eluate was stored at -80^{0}C. 10 µl of extracted RNA was used to amplify the Pol gene (**Reverse-trancriptase and Protease regions**) of HIV-1 using the one step superscript III Hifi RT-PCR kit (Invitrogen) as suggested by manufacture's protocol. 10µM of the forward primer POLF1 (5'-TGAARGAZTGYACTGARAGRCAGGCTAAT-3') and 10µM of the reverse primer POLR1 (5'-CCTCZTTYTTGCTAYTTYCCTGTT-3') were used for the RT-PCR under the following thermocycling conditions: (40^{0}C for 30min, 94^{0}Cfor 2min, [94^{0}C for 30s, 55^{0}C for 30s, extension 68^{0}C for 1.30min for 35 cycles], final extension at 68^{0}C for 7min and hold at 4^{0}C) using the Gene Amp PCR system 9700 thermocycler from applied biosystems.

Nested PCR was done using 2 µl of the primary PCR product and platinum taq high fidelity enzyme kit (Invitrogen). 10µM of the forward primer (5'- CTTTARYTTCCCTCARATCATCT -3') and 10µM of the reverse primer POLR2 (5'- GGCTCTTGATAAATTTGATATGTCCAT-3') were used for the nested PCR under the following thermocycling conditions; (at 94^{0}C for 2min, [94^{0}C for 30s, 55^{0}C for 30s, extension at 68^{0}C for 1min for 35 cycles], final extension at 68^{0}C for 7min and 4^{0}C hold). The correct size of amplified product (1.3kb) was visualized using agarose gel electrophoresis using a 1% agarose gel stained with red safe (0.5µg/ml) under a UV transilluminator. All the primers (Kaleebu et al., 2015) are shown in appendix C.

Library preparation and sequencing for the detection and quantification of minority resistant variants

The amplified PCR product of HIV-1 Pol gene (1.3kb) from the protease (PR) and reverse transcriptase (RT) regions was cleaned using the Qiagen purification kit (Qiagen, German) using the following procedure: A 5x volume of buffer PBI was added to 1x volume of sample and vortexed for 15 seconds. The mixture was then transferred to the Qiaquick spin column and centrifuged for one minute at 13000 rpm. The Qiaquick spin column was transferred into a new collection tube. Buffer PE (750 µl) was added onto the Qiaquick spin column and centrifuged for one minute at 13000 rpm. The Qiaquick spin column was then placed into a new 1.5ml micro-centrifuge tube. EB buffer was added into the spin column and spun at 13000 rpm to get an eluate which was stored at -20^{0}C. The cleaned PCR amplicons were quantitated using the Qubit fluorometer (Invitrogen Thermoscientific) and the qubit ds DNA HS Assay kit according to the manufacturer's instructions as follows; The PCR amplicons (10 µl) were pipetted into a clean 96-well plate and diluted using nuclease free water (90 µl) while mixing up and down. Using qubit assay tubes, qubit standards were prepared by adding10 µl of standards to 190 µl of Working Solution. The diluted samples were also prepared by adding 5 µl diluted sample to195 µl of Working Solution. The mixtures were vortexed and incubated at room temperature for 3 minutes. Using the qubit fluorometer, the lower and higher standards were measured. The sample concentration was also measured and recorded on a worksheet, and it lay between the lower and higher qubit standards. The samples were diluted to a final concentration of 0.2ng/µl. Sequencing libraries were prepared using the Nextera XT DNA library preparation kit (Illumina, SanDieogo, CA, USA). The process of library preparation included tagmentation, indexing, purification, and library normalization. Tagmentation randomly fragments the PCR amplicon, and simultaneously tags the DNA with adaptor sequences. Using the Nextra XT library preparation kit, the diluted sample (0.2ng/ µl), was pipetted into a new 96-well midi plate (5 µl). Tagment DNA buffer (10 µl) was added into the midi plate and the Amplicon tagment buffer (5 µl) was also added into the same plate. The midi plate was then vortex mixed and spun down at 280×g for one minute. This was placed into the 9700 Applied biosystems thermocycler under the following conditions [(55^{0}C for 5min and hold at 10^{0}C)]. The samples were removed from the thermocycler and incubated for 5 minutes at room temperature (25^{0}C). Immediately after tagmentation, index PCR was done to append sample specific indexes to each fragment and create a library of various sized fragments

sharing specific dual indexes. Indexes from the Nextera index kit were arranged on the truseq plate as shown below (Figure S2).

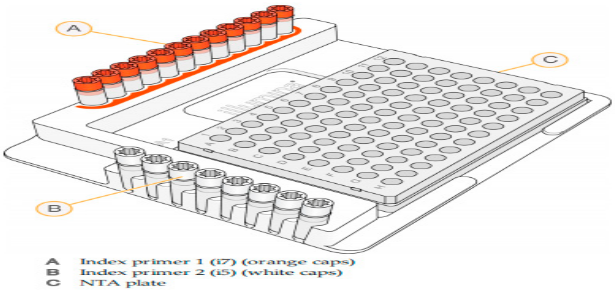

Figure S2: Illustration of an index PCR tray

The Nextera PCR master mix (NPM) was pipetted into the 96 well plate (15µl). Then the index primers i7 and i5 (5 µl) were added to the plate by row and by column respectively. The mixture was pipetted up and down and a brief spin at 280xg was done. Index PCR was done under the following thermos cycling conditions (Table S1).

Table S1: PCR steps and the Thermocycler conditions used

**Step**	**Temp** °C	**Time**	
Tagment Enzyme Inactivation	65	3 min	
Denaturation and Activation	95	30 sec	
PCR Cycles	95	10 sec	×35
	55	30 sec	
	68	30 sec	
Final Extension	68	5 min	

After index PCR, library purification was done as follows; index PCR product (50µl) was placed into a new midi plate. Angencourt AMpure beads (50µl) from Beckman coulter were added to the

index product and vortexed for two minutes at 1800rpm. The midi plate was then put on to the magnetic stand and left on for two minutes. The supernatant was pipetted off and 200 µl of ethanol (80%) was dispensed into the plate. This step was repeated three times until all the ethanol was removed. The plate was left to air dry for 15 minutes off the magnet and resuspension buffer (52.5 µl) was added to the plate thereafter while mixing up and down. The mixture was incubated for 2 minutes at room temperature. Thereafter it was placed on the magnetic stand for two minutes. The supernatant (45 µl) was pipetted into a new 96-well plate. To ensure equal library representation during sequencing, library normalization was done using the Nextera library normalization kit to obtain a 10-12pM library with inserts that are 500-1000 base pairs. The index PCR amplicon (20 µl) was pipetted into a new well labelled 96-well midi plate. Library Normalization Additives (4.4ml) was placed into a 50 ml conical tube together with the library normalization beads (800 µl) and mixed well. This mixture (45 µl) was transferred into the midi plate containing the index amplicons. The midi plate was sealed and shaken on a microplate shaker at 1,800 rpm for 30 minutes. After 30 minutes shaking, the MIDI plate was placed on the magnetic stand for 2 minutes to allow the beads to pellet. While the MIDI plate was still on the magnetic stand, the supernatant was removed and discarded. The MIDI plate was removed from the magnetic stand and Library Normalization Wash 1(45 µl) was added into each of the wells. The plate was sealed and shaken on a microplate shaker at 1,800 rpm for 5 minutes. The plate was then placed on to the magnetic stand for 2 minutes to allow the beads to pellet. The supernatant was removed and discarded. A second wash was repeated, and the MIDI plate was removed from the magnetic stand. 0.1 N NaOH (30 µl) was aliquoted into each well and the plate was sealed and shaken on a microplate shaker at 1,800 rpm for 5 minutes. After the five minutes' incubation, the MIDI plate was placed on the magnetic stand for 2 minutes. The supernatant (30 µl) from the midi plate was removed and added to a new well labelled 96 well plate containing Library Normalization Storage Buffer (30 µl). using a multi-channel pipet, the mixture was mixed up and down and centrifuged at 1800 rpm. This is the normalised library of 96 samples. In preparation for the Miseq run, a sample sheet was prepared on the Miseq. The flow cell (Figure S3) was then washed with laboratory grade water to remove all the salts that would inhibit sequencing and there after air dried.

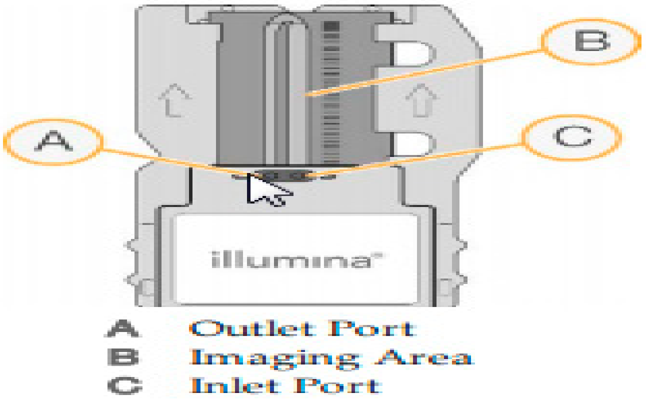

Figure S3: The illustration of the flow cell

The normalized libraries (5µl) were pooled into a 1.5ml eppendorf tube and mixed thoroughly. From the pool, 24 µl of pooled libraries were added to Hybridization buffer (576 µl) from the Miseq kit (V2 500-cycle) from Illumina, USA. This was incubated in heat block at 96^0C for two minutes. From the heating block, the mixture was incubated for five minutes on ice cold water. Phix control (20pM) from the phix kit was spiked at 20% in the pooled amplicons. These were loaded on to the cartridge and loaded into the Miseq for running. In total, 167 samples were loaded on the Miseq in three runs.
